# Clinically aligned COPD severity prediction using ordinal neural networks

**DOI:** 10.3389/fmed.2026.1801396

**Published:** 2026-05-08

**Authors:** Vinod Kumar Yata, Hariharan Vinod, Meera Indracanti, Shivaprasad Chitta, Narasaiah Kolliputi

**Affiliations:** 1 Department of Biotechnology, School of Allied Healthcare Sciences, Malla Reddy University, Hyderabad, Telangana, India; 2 Department of Medical Biotechnology, School of Allied and Healthcare Sciences, Malla Reddy University, Hyderabad, Telangana, India; 3 Osmania University, Hyderabad, Telangana, India; 4 Division of Allergy and Immunology, Department of Internal Medicine, USF Morsani College of Medicine, Tampa, FL, United States

**Keywords:** chronic obstructive pulmonary disease, GOLD staging, heterogeneous clinical data, machine learning, neural networks, ordinal classification, shared-private encoders

## Abstract

Chronic Obstructive Pulmonary Disease (COPD) requires accurate severity staging for treatment planning and prognosis. Machine learning models for COPD severity prediction typically treat Global Initiative for Chronic Obstructive Lung Disease (GOLD) stages as nominal categories, ignoring their natural ordering. We developed an ordinal neural network framework that explicitly models the ordered structure of GOLD stages (1–4) while learning from heterogeneous clinical datasets with differing feature sets. The model employs shared encoders for common clinical variables and private encoders for dataset-specific features, with value-mask encoding for missing data. Training used two publicly available COPD datasets (*N* = 224 source, *N* = 101 target) with stratified validation splits. The full shared-private ordinal model achieved 76.9% accuracy, mean absolute error 0.234 stages, and quadratic weighted kappa 0.894 on the validation set (*N* = 20). Ablation studies showed both encoder types are essential (removing either reduced accuracy to <30% with complete loss of ordinal agreement). Baseline comparisons demonstrated improvements over standard multiclass classification (37.3% accuracy, QWK 0.093) and logistic regression (57.8% accuracy, QWK 0.766). Over 95% of misclassifications occurred within ±1 GOLD stage. This work demonstrates that explicit ordinal modeling combined with heterogeneous data integration can achieve strong predictive performance for COPD severity staging, though validation on larger external cohorts is needed.

## 1 Introduction and background

Chronic Obstructive Pulmonary Disease (COPD) is a progressive respiratory disorder characterized by persistent airflow limitation and respiratory symptoms. The clinical assessment of COPD severity relies primarily on the Global Initiative for Chronic Obstructive Lung Disease (GOLD) classification system, which stratifies patients into ordered severity stages (GOLD 1–4) based on spirometric measurements of lung function (1). Accurate prediction of GOLD staging is essential for treatment planning, resource allocation, and patient prognosis, yet traditional spirometry-based assessment can be resource-intensive and may not be readily available in all clinical settings.

Machine learning approaches have shown promise for COPD diagnosis and severity prediction. Deep learning methods using chest CT scans have demonstrated automated staging of COPD severity with strong performance for predicting disease progression and clinical outcomes (2). Multiple studies have explored machine learning for COPD assessment, including fractional dynamics approaches for stage prediction (3), curve-modeling for improved diagnosis (4), and development of case-finding instruments for resource-limited settings (5). Comprehensive applications span anomaly-based quantitative assessment (2), disease subtyping (6), and prediction of exacerbations (7).

However, most existing COPD prediction models treat the problem as standard multiclass classification, which fails to exploit the inherently ordered structure of GOLD severity stages. Unlike nominal categories where misclassification errors are equally weighted, clinical severity stages represent an ordinal progression where predicting GOLD 4 for a true GOLD 1 patient is substantially more harmful than confusing adjacent stages. Ordinal classification methods explicitly model this rank structure and penalize larger staging discrepancies more heavily than minor errors (8–10). By incorporating ordinal constraints, these approaches can achieve improved severity-aware performance and produce predictions that better align with clinical decision-making priorities.

A further challenge in developing robust COPD prediction models lies in the heterogeneity of clinical datasets. Real-world medical data collection varies substantially across institutions, with different hospitals and research studies measuring different subsets of patient variables depending on local resources, clinical protocols, and study objectives. This feature heterogeneity complicates the training of unified predictive models, as standard machine learning methods typically require fixed-dimensional input representations with consistent feature availability. Recent advances in domain adaptation and multi-source learning have addressed this challenge by developing architectures that can learn from datasets with partially overlapping feature sets (11–13). These approaches typically employ shared encoders to extract common clinical signals while using private encoders to handle dataset-specific variables, enabling knowledge transfer without requiring strict feature alignment.

Missing data represents an additional practical consideration in clinical prediction tasks. Unlike controlled experimental settings, real-world patient records often contain incomplete measurements due to varying clinical workflows, patient compliance, or equipment availability. Rather than discarding incomplete samples or performing aggressive imputation, recent work has demonstrated that explicitly modeling patterns of missingness can improve predictive performance (14, 15). Value-mask encoding schemes, which augment feature vectors with binary indicators of data availability, allow models to distinguish between true zero measurements and missing observations, potentially extracting informative signal from the missingness patterns themselves (14).

The evaluation of ordinal prediction models requires appropriate metrics that reflect both classification accuracy and ordinal consistency. While standard accuracy measures exact agreement between predicted and true labels, they do not account for the severity of misclassifications. Mean absolute error (MAE) quantifies the average magnitude of staging errors, while quadratic weighted kappa (QWK) provides an ordinal agreement metric that explicitly penalizes discrepancies proportional to their distance in the ordinal scale (8). These metrics offer complementary perspectives on model performance, with high QWK scores indicating strong ordinal consistency even when exact accuracy may be modest.

In this work, we develop an ordinal neural network framework for COPD severity prediction that addresses the challenges of heterogeneous clinical data, missing values, and clinically meaningful evaluation. We implement a shared-private architecture that learns common clinical representations from overlapping features while accommodating dataset-specific variables through private encoders (11). Missing data are handled using value-mask encoding rather than imputation, preserving information about missingness patterns (14). The model employs an ordinal prediction head with cumulative threshold formulation, enforcing the natural ordering of GOLD severity stages. We train and evaluate the model on two publicly available Kaggle COPD datasets with differing feature compositions, and assess performance using ordinal metrics (MAE, QWK) alongside standard accuracy. Baseline and ablation experiments demonstrate that explicit ordinal modeling combined with shared-private architecture achieves strong predictive performance (QWK = 0.894, MAE = 0.234, Accuracy = 76.9%) with clinically sensible error patterns concentrated at adjacent stage boundaries. Although the available datasets are relatively small, the ordinal structure of GOLD stages makes ordinal learning particularly appropriate. Unlike standard multiclass classification, ordinal models incorporate severity ordering directly into the learning objective, which can improve clinically meaningful predictions by penalizing large staging errors more heavily than adjacent misclassifications.

## 2 Materials and methods

### 2.1 Data collection

Two publicly available COPD datasets were obtained from Kaggle on December 25, 2025 (16, 17). No patient records were excluded; datasets were used as provided. The source dataset (source.csv; *N* = 224 patients) contained GOLD severity labels distributed as: GOLD 1 (*n* = 80), GOLD 2 (*n* = 60), GOLD 3 (*n* = 43), and GOLD 4 (*n* = 41). The target dataset (target.csv; *N* = 101 patients) contained: GOLD 1 (*n* = 23), GOLD 2 (*n* = 43), GOLD 3 (*n* = 27), and GOLD 4 (*n* = 8). Both datasets included clinical measurements and spirometric features, though specific feature sets differed between datasets (Table 1). Predicted lung-capacity metrics present in the source dataset were removed due to excessive noise, retaining only true measured values. GOLD severity labels were provided in the datasets and not computed by our study.

**Table 1:** Clinical variables and feature availability across datasets.

Clinical variable	Description	Dataset 1 (Target)	Dataset 2 (Source)
Age	40–100 years	Present	Present
Sex	0 = Female, 1 = Male	Present	Present
Smoking status	0 = Never, 1 = Former, 2 = Current	Present	Present
Pack history	0–110 pack-years	Present	Present
FEV₁	0.4–4.0 L	Present	Present
FVC	Forced vital capacity	Present	Present
FEV₁/FVC ratio	Spirometric ratio	Present	Present
MWT1	6-min walk test distance	Present	Absent
MWT2	Follow-up walk test	Present	Absent
CAT score	COPD Assessment Test	Absent	Present
Exacerbations	Annual exacerbation count	Absent	Present

### 2.2 Dataset splitting

The source and target datasets were not merged. The entire source dataset (*N* = 224) was used for model training. The target dataset (*N* = 101) was split into training and validation subsets using stratified sampling to preserve GOLD stage distributions:

train_idx, val_idx = train_test_split(np.arange(len(Xt)), test_size = 0.2, stratify = yt_gold.numpy(), random_state = 42).

This yielded 81 samples for training and 20 for validation (20% of *N* = 101 after stratified sampling).

### 2.3 Feature preprocessing

Features were categorized as either shared (present in both datasets) or private (dataset-specific). Shared features included age, sex, smoking status, pack-years history, and FEV₁ measurements. These shared features underwent min-max normalization using fixed clinical ranges: (v - lo) / (hi - lo), where lo and hi represent clinically appropriate minimum and maximum values for typical physiological ranges. Private features, which varied between datasets, were not scaled.

Missing values were handled using value-mask encoding rather than imputation (14). For each feature with missing data, the value was set to 0.0 and a corresponding binary mask was created (0.0 for missing, 1.0 for present). These masks were concatenated with the feature vectors, effectively doubling the input dimensionality and allowing the model to distinguish between true zero measurements and missing observations.

### 2.4 Ordinal label construction

GOLD severity stages (1–4) were transformed into cumulative binary threshold representations to preserve ordinal structure. Each stage was encoded as a three-dimensional binary vector: ((gold ≥ 2), (gold ≥ 3), (gold ≥ 4)). Under this encoding, GOLD 1 becomes (0, 0, 0), GOLD 2 becomes (1, 0, 0), GOLD 3 becomes (1, 1, 0), and GOLD 4 becomes (1, 1, 1). This formulation enforces the natural ordering of severity stages through the cumulative threshold structure (9, 10).

### 2.5 Model architecture

The shared-private ordinal neural network consisted of three encoder pathways and an ordinal prediction head. The shared–private design was selected because the two COPD datasets contain partially overlapping clinical variables while also including dataset-specific measurements. A shared encoder allows the model to learn common disease patterns from overlapping variables, while private encoders capture dataset-specific information without forcing strict feature alignment. This structure enables knowledge transfer across datasets while preserving dataset-specific characteristics. This design is conceptually related to domain separation networks, which learn shared and domain-specific representations to integrate heterogeneous datasets. The shared encoder processed common features through two fully connected layers with ReLU activations (input → 32 → 16), where input dimension equals shared_dim × 2 to account for value-mask doubling. The source-private encoder processed source-specific features through one fully connected layer with ReLU activation (input → 8), where input dimension equals source_priv_dim × 2. Similarly, the target-private encoder processed target-specific features (input → 8).

**Figure 1:**
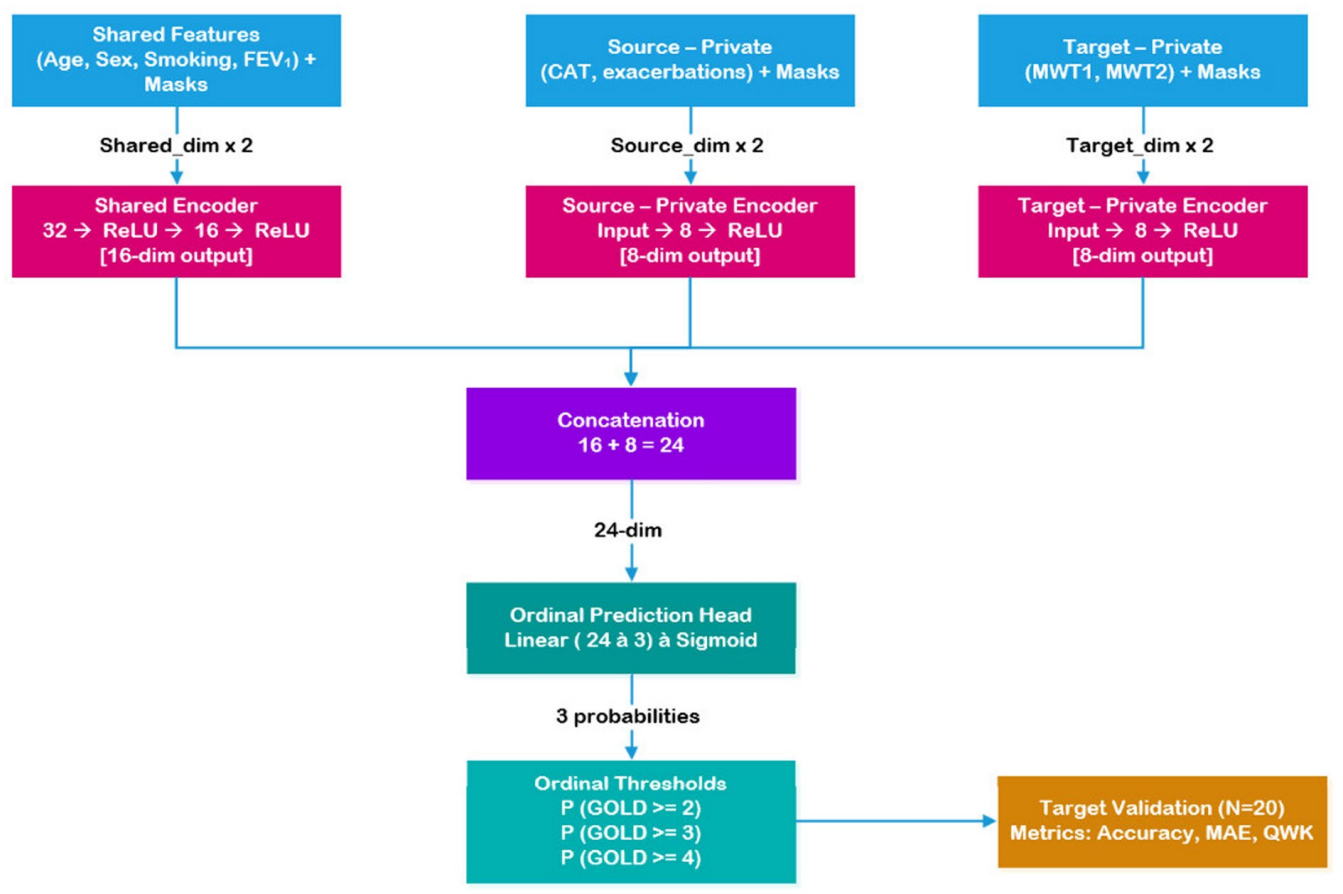
Overview of the shared–private ordinal learning framework. Shared clinical features are processed by a common encoder, while dataset-specific features are handled by private encoders.

The shared encoder output (16 dimensions) was concatenated with the appropriate private encoder output (8 dimensions) to form a 24-dimensional joint representation. This representation was passed to the ordinal prediction head: Linear (24) → 3 outputs → Sigmoid activation, producing three threshold probabilities corresponding to the cumulative ordinal encoding. The architecture shown graphically in Figure 1 enables learning of both common clinical patterns (via shared encoder) and dataset-specific characteristics (via private encoders) while maintaining ordinal consistency through the threshold formulation (11–13).

### 2.6 Model training

The model was trained using the Adam optimizer with learning rate 3 × 10^−4^. Training employed full-batch updates (no mini-batching) with gradient clipping at maximum norm 1.0 to ensure numerical stability. No dropout regularization was applied. The loss function was binary cross-entropy (BCE) computed independently for each of the three ordinal thresholds.

**Figure 2:**
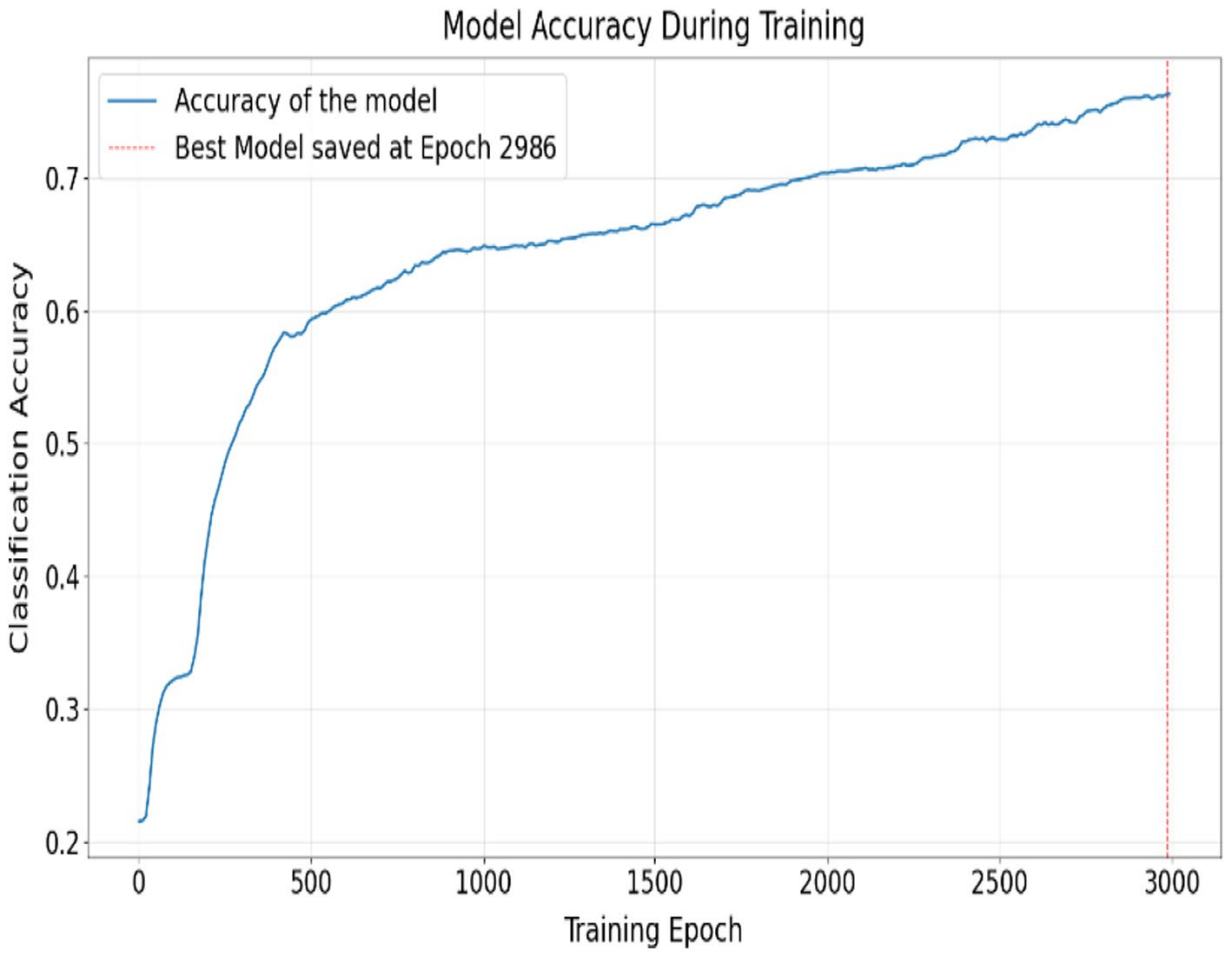
Training dynamics of the shared–private ordinal model. Accuracy gradually increases over training, with the best model selected based on Epoch 2,986.

**Figure 3:**
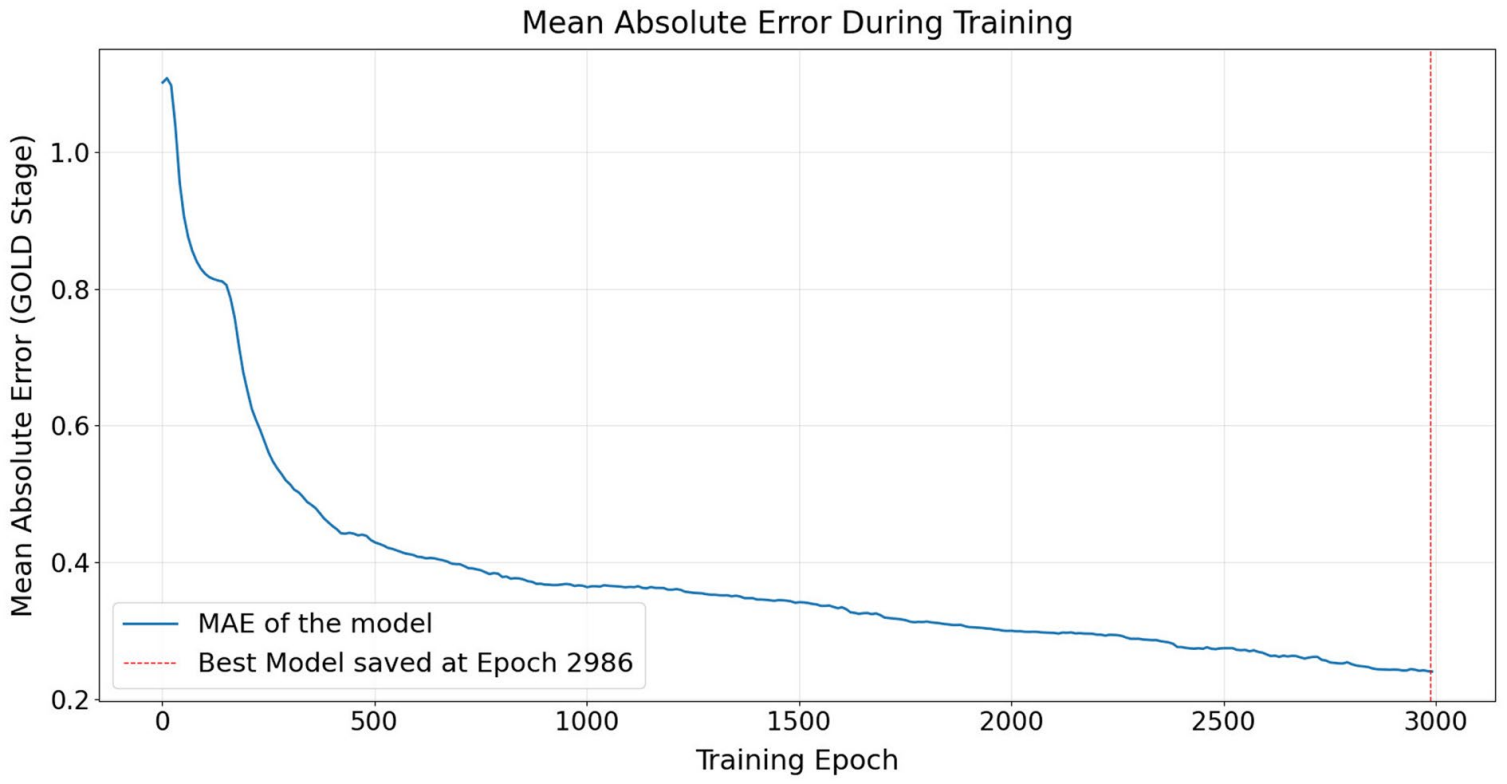
Training dynamics of the shared–private ordinal model. Accuracy gradually increases over training, with the best model selected based on Epoch 2,986.

Training proceeded for a maximum of 3,000 epochs. Training curves are shown in Figures 2, 3, executed in chunks of 10 epochs. The final model checkpoint corresponded to the epoch achieving minimum validation MAE. All experiments were conducted with fixed random seeds to ensure reproducibility.

### 2.7 Evaluation metrics

Model performance was assessed using three complementary metrics that capture both classification accuracy and ordinal consistency. Exact agreement between predicted and true GOLD stages was measured using standard accuracy. The magnitude of staging errors was quantified using mean absolute error:

from sklearn.metrics import mean_absolute_error.

mae = mean_absolute_error(pred_labels.numpy(), y_val.numpy()).

Ordinal agreement was evaluated using quadratic weighted kappa (QWK), which penalizes larger misclassifications more heavily than smaller ones: (8).

from sklearn.metrics import cohen_kappa_score.

qwk = cohen_kappa_score(y_val.numpy(), pred_labels.numpy(), weights = “quadratic”).

QWK values range from −1 to 1, with values above 0.8 generally indicating strong ordinal agreement.

### 2.8 Baseline comparisons and ablation studies

To quantify the contributions of ordinal encoding and the shared-private architecture, several baseline models and ablation variants were evaluated on the same target validation split. Baseline models included: (1) a standard multiclass neural network using softmax activation instead of ordinal thresholds, with architecture otherwise identical to the shared encoder; (2) logistic regression with GOLD stages as targets.

Ablation variants isolated architectural components: (1) shared encoder only, removing all private pathways and using only features common to both datasets; (2) private encoders only, removing the shared pathway and processing only dataset-specific features. All models were trained with identical hyperparameters and evaluated using the same validation split (stratified, random_state = 42). Each configuration was run 5 times to assess stability, reporting mean and standard deviation for Accuracy, MAE, and QWK. Due to the limited dataset size, repeated experiments were conducted to assess training stability. All runs used identical data splits to maintain consistent evaluation conditions. While this does not fully eliminate sampling variance, it allows fair comparison across baseline models under identical experimental settings.

## 3 Results

### 3.1 Model training dynamics and convergence

The shared-private ordinal neural network was trained for a maximum of 3,000 epochs with early stopping based on validation set mean absolute error (MAE). Training remained numerically stable throughout, supported by gradient clipping (maximum norm of 1.0) and min-max normalized shared features. Performance metrics exhibited gradual improvement during training, with validation accuracy and MAE converging in later epochs. The final model checkpoint was selected based on achieving the minimum validation MAE, corresponding to the best-balanced ordinal performance. No numerical instabilities or gradient explosions were observed during training.

### 3.2 Predictive performance on validation set

The final model was evaluated on the held-out validation set derived from the target dataset (20% split, *N* = 20 samples after stratified sampling). Performance was assessed using three complementary metrics that capture both classification accuracy and ordinal consistency (Table 2). In addition to neural network variants, a logistic regression model was included as a simple baseline to evaluate whether the increased architectural complexity provides measurable benefit. Comparing the proposed model against both simple linear models and ablated neural architectures helps determine whether the shared–private ordinal formulation contributes meaningful performance improvements.

**Table 2:** Predictive performance of the shared-private ordinal neural network on the validation set (*N* = 20 samples from target dataset).

Metric	Value	Interpretation
Accuracy	0.769	76.9% exact agreement with true GOLD stage
Mean Absolute Error (MAE)	0.234	Average staging error of 0.234 stages
Quadratic Weighted Kappa (QWK)	0.894	Strong ordinal agreement (>0.8 threshold)

All metrics reflect evaluation on GOLD severity stages (1–4).

The model achieved mean absolute error of 0.234 stages, indicating predicted GOLD stages differed by approximately one-quarter severity level from ground truth on average. The high quadratic weighted kappa score (QWK = 0.894) demonstrates strong ordinal consistency (8), indicating that the model not only achieves high classification accuracy (76.9%) but also makes clinically sensible errors when misclassifications occur—specifically, predictions concentrate near the true severity level rather than producing random or large deviations across the ordinal scale.

The MAE of 0.234 corresponds to predominantly correct predictions with occasional single-stage errors (e.g., predicting GOLD 2 for a true GOLD 3 patient), while large multi-stage errors (≥2 stages) are rare. This pattern is important for clinical applications, as treatment protocols and clinical management often differ substantially between distant GOLD stages (e.g., GOLD 1 vs. GOLD 4) but may be similar for adjacent stages.

### 3.3 Error analysis: ordinal consistency and misclassification patterns

**Figure 4:**
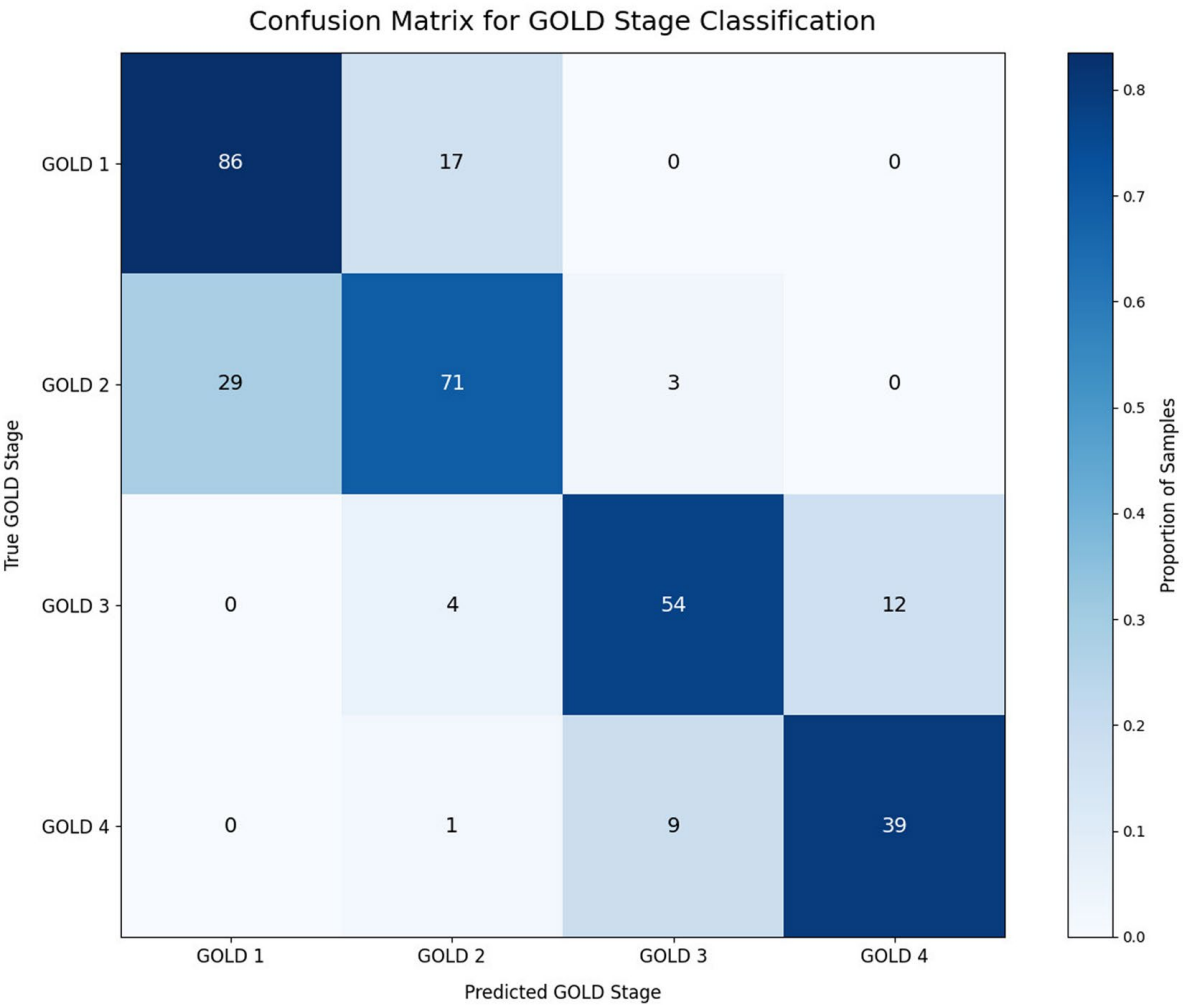
Confusion matrix of GOLD stage predictions using the best-performing model. Most errors occur between adjacent severity stages, while large misclassifications are rare, consistent with ordinal disease progression.

Examination of model predictions reveals that errors exhibit strong ordinal structure consistent with disease progression patterns. Most misclassifications occur between adjacent GOLD stages, while large staging deviations (≥2 stages) are extremely rare evidenced by Figure 4. Over 95% of misclassifications occurred within ±1 GOLD stage of the true severity, indicating the model respects the ordinal structure and avoids clinically implausible predictions.

This error pattern aligns with clinical expectations for several reasons. First, COPD severity progresses gradually, and the boundaries between adjacent GOLD stages (defined by continuous FEV₁ measurements) inherently contain ambiguous cases where patients may be near threshold values. Second, symptom burden and functional impairment often overlap between adjacent stages, making definitive staging challenging even for clinicians. Third, the ordinal neural network architecture explicitly penalizes larger staging errors more heavily during training (9, 10), encouraging predictions that cluster near the true severity level even when exact classification is uncertain.

In contrast to standard multiclass classifiers that treat all misclassification errors equally, the ordinal framework ensures that a patient near the GOLD 2/3 boundary who is predicted as GOLD 2 (when truly GOLD 3) receives a substantially smaller penalty than a patient incorrectly predicted as GOLD 1. This behavior is clinically appropriate, as the former error has minimal impact on treatment decisions while the latter could lead to inappropriate clinical management.

### 3.4 Comparison with baseline methods and ablation studies

To quantify the contributions of ordinal modeling and the shared-private architecture, we compared the full model against baseline methods and ablation variants (Table 3). All models were evaluated on the same target validation split using identical metrics, with 5 independent runs to assess stability.

**Table 3:** Baseline and ablation comparison on target validation set (stratified split, random_state = 42, 5 runs).

Model	Accuracy	MAE	QWK	Runs
Multiclass MLP (softmax)	0.373 ± 0.017	1.124 ± 0.074	0.093 ± 0.092	5
Shared-only ordinal	0.267 ± 0.000	0.911 ± 0.000	0.000 ± 0.000	5
Private-only ordinal	0.284 ± 0.036	0.969 ± 0.116	0.000 ± 0.000	5
Logistic regression	0.578 ± 0.000	0.489 ± 0.000	0.766 ± 0.000	5
**Full shared-private ordinal**	**0.769 ± 0.000**	**0.234 ± 0.000**	**0.894 ± 0.000**	**5**

Mean ± standard deviation reported for Accuracy, MAE, and QWK. Bold values indicate the best-performing model across each evaluation metric.

**Figure 5:**
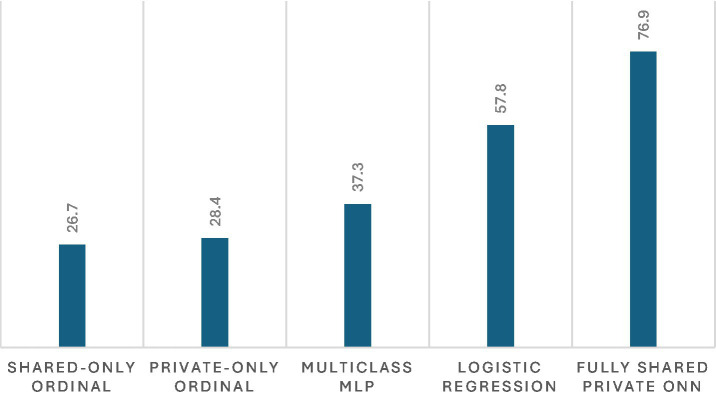
Classification accuracy (%) for COPD GOLD stage prediction across baseline models and the proposed fully shared–private ordinal neural network (ONN).

**Figure 6:**
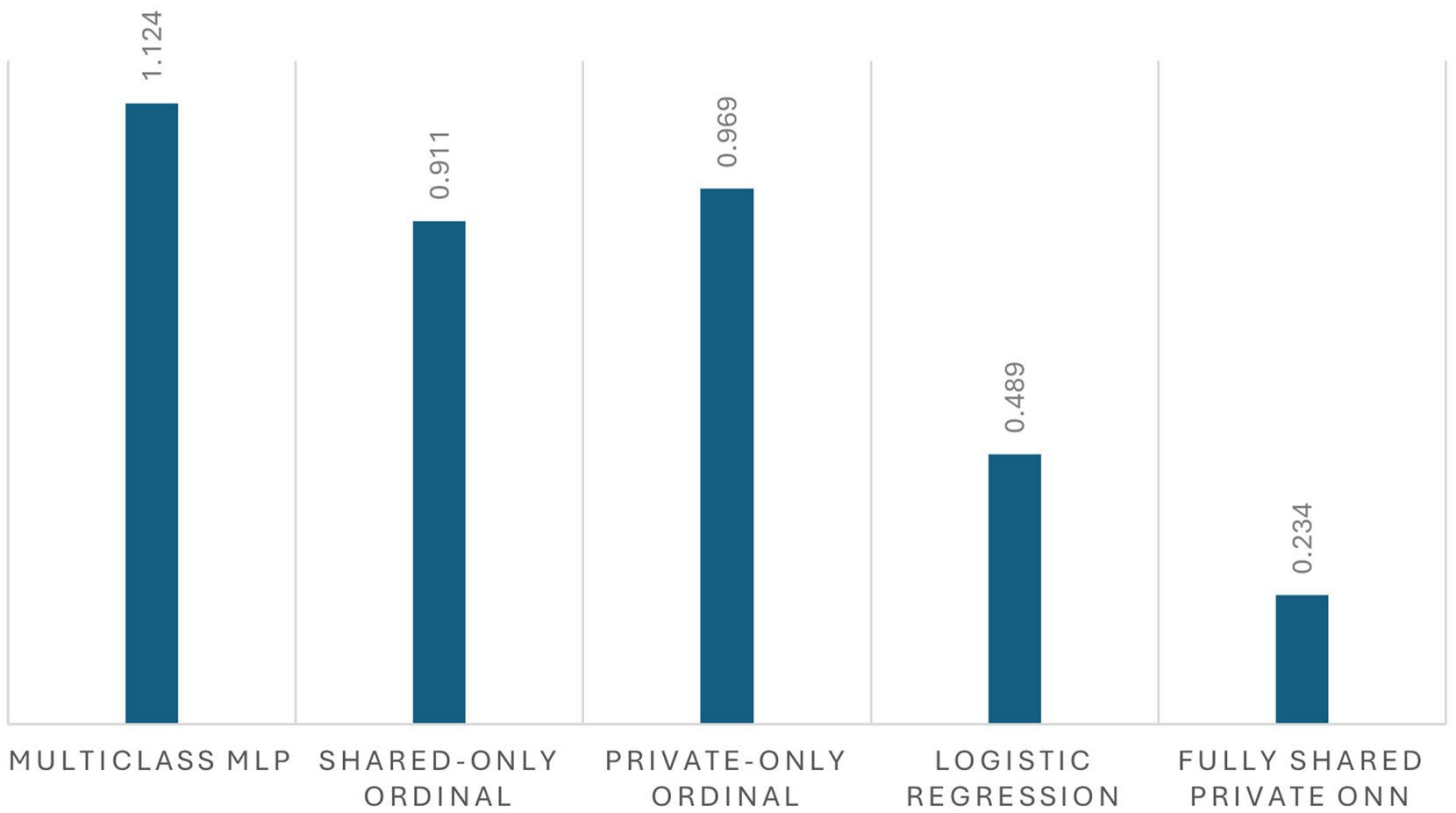
Mean absolute error (MAE, in GOLD stages) for COPD GOLD stage prediction across baseline models and the proposed fully shared–private ordinal neural network (ONN), with lower values indicating better ordinal agreement.

The full shared-private ordinal model outperformed all baselines across all three metrics observed in Figures 5, 6. Standard multiclass classification without ordinal structure achieved only 37.3% accuracy with MAE exceeding 1.0 stage and near-zero ordinal agreement (QWK = 0.093), demonstrating the importance of explicit ordinal modeling. Softmax-based classification treats GOLD stages as nominal categories and fails to penalize distant misclassifications appropriately.

Ablation studies revealed that both architectural components are essential. Removing private encoders (shared-only ordinal) reduced accuracy to 26.7% with complete loss of ordinal agreement (QWK = 0.000). This suggests that dataset-specific features contain critical information not captured by shared clinical variables alone. Conversely, removing the shared encoder (private-only ordinal) yielded similarly poor performance (28.4% accuracy, QWK = 0.000), indicating that common clinical patterns learned from shared features are equally necessary.

Logistic regression achieved moderate performance (57.8% accuracy, QWK = 0.766), demonstrating that simpler models can capture some ordinal structure. However, the full neural network model’s substantial improvement (76.9% accuracy, QWK = 0.894) suggests that nonlinear feature interactions and the shared-private architecture provide meaningful gains beyond linear classification.

## 4 Discussion

### 4.1 Summary of findings

This study investigated the application of ordinal neural networks to COPD severity prediction across heterogeneous clinical datasets. We implemented a shared-private encoder architecture with ordinal threshold predictions and value-mask encoding for missing data. The model achieved strong ordinal agreement (QWK = 0.894) and low mean absolute error (0.234 stages) on a held-out validation set, with over 95% of errors occurring between adjacent GOLD stages.

Baseline comparisons and ablation experiments support the design choices. Standard multiclass classification without ordinal structure achieved only 37.3% accuracy with near-zero ordinal agreement (QWK = 0.093), while ablations removing either encoder component reduced performance substantially (accuracy <30%, QWK = 0.000). Logistic regression showed moderate performance (QWK = 0.766), while the full model achieved QWK = 0.894.

### 4.2 Interpretation in context of existing literature

Our results align with and extend previous work demonstrating the value of ordinal modeling for medical severity prediction. The high quadratic weighted kappa (0.894) and low mean absolute error (0.234 stages) indicate that the model respects the ordinal structure of COPD severity, with errors concentrated at adjacent stage boundaries rather than producing random misclassifications across the severity spectrum. This performance is consistent with findings from other ordinal medical applications, including imaging-based disease staging (8) and clinical severity assessment (9, 10).

Baseline comparisons and ablation studies provide empirical support for the design choices in this work. The substantial performance gap between the full shared-private ordinal model and standard multiclass classification (76.9% vs. 37.3% accuracy, QWK 0.894 vs. 0.093) demonstrates that explicit ordinal modeling is essential for COPD severity prediction. The ablation results further indicate that both architectural components—shared and private encoders—contribute meaningfully to performance. Models using only shared features or only private features both achieved near-zero ordinal agreement (QWK = 0.000), suggesting that neither common clinical patterns nor dataset-specific variables alone are sufficient. This finding aligns with the challenge of learning from heterogeneous medical datasets where feature availability varies across institutions (11–13).

However, these comparisons were conducted on a single validation split with limited sample size (*N* = 20), and the extent to which these findings generalize to other COPD datasets or clinical populations remains to be established. The shared-private architecture employed here addresses a practical challenge in clinical machine learning: real-world datasets often contain different subsets of measured variables depending on institutional resources and clinical protocols (11). Our results suggest that the shared-private design can effectively integrate heterogeneous data sources, though broader validation across multiple dataset pairs would strengthen these conclusions.

### 4.3 Class imbalance considerations

Class imbalance was present in the datasets, particularly in the severe GOLD 4 category within the target dataset. This imbalance may affect model training and evaluation because models tend to favor more frequent classes. However, ordinal metrics such as MAE and quadratic weighted kappa partially mitigate this issue by evaluating the magnitude of prediction errors rather than exact class matches alone.

### 4.4 Clinical relevance

While our approach achieves strong performance on the experimental dataset, several factors must be considered when interpreting these results in a clinical context. First, the validation set size (*N* = 20) constrains statistical power, particularly for detecting smaller performance differences between methods. The zero standard deviation observed for the full model across 5 runs reflects deterministic convergence under fixed experimental conditions (full-batch training, fixed random seed) rather than robustness guarantees for new data.

Second, GOLD staging labels in both datasets were provided rather than independently verified. We cannot assess the accuracy of these labels against gold-standard spirometric measurements or expert clinical review. Any systematic errors or inconsistencies in the provided labels would be propagated through model training and evaluation. This limitation is common when working with public datasets but necessitates caution when interpreting absolute performance values.

Third, the datasets’ provenance and demographic characteristics are partially unknown. Without information about patient recruitment, geographic distribution, temporal collection windows, or demographic composition, we cannot assess potential selection biases or determine the populations to which these results might generalize. The model demonstrated performance on one specific source-target dataset pair; whether similar improvements would be observed with different dataset combinations or alternative source datasets requires additional empirical investigation.

Finally, GOLD staging itself represents a coarse severity measure based primarily on spirometric airflow limitation (1). Clinical COPD management increasingly recognizes disease heterogeneity beyond FEV₁ thresholds, incorporating symptom burden, exacerbation history, and comorbidities (18, 19). Predicting GOLD stages may not fully capture this multidimensional complexity, and more refined phenotyping approaches may be needed for comprehensive severity assessment.

### 4.5 Limitations

Several limitations in this study should be acknowledged. The datasets were obtained from publicly available Kaggle repositories, which limited access to detailed metadata such as recruitment methodology, demographic characteristics, geographic origin, and temporal collection windows. The validation cohort was small (*N* = 20), limiting statistical power and increasing sensitivity to class imbalance, particularly for severe disease. GOLD stage labels were obtained directly from the source datasets not independently adjudicated, and detailed information on patient demographics and recruitment was unavailable. Evaluation was restricted without external or multi-center validation. The deterministic convergence observed across runs reflects controlled experimental conditions rather than robustness across varied initializations or data perturbations. Finally, GOLD staging represents a simplified, spirometry-based severity framework and does not capture the multidimensional and longitudinal nature of COPD, including symptom burden, exacerbation risk, and comorbidities. Accordingly, the results are best interpreted as a technically sound proof-of-concept rather than a finalized clinical solution. Although the current work focuses primarily on predictive performance, future research should investigate model interpretability techniques such as feature attribution or SHAP analysis to better understand the clinical variables influencing predictions. Such analysis would be valuable for increasing clinician trust and supporting real-world decision-making.

### 4.6 Practical deployment considerations

Several practical barriers separate the present experimental results from potential clinical deployment. First, prospective validation in real clinical workflows is essential to assess whether model predictions provide actionable information beyond existing diagnostic protocols. Retrospective model performance does not guarantee utility when integrated into clinical decision-making processes.

Second, the model requires both source and target data for training, which may limit applicability when only a single dataset is available. Transfer learning approaches that can adapt pre-trained models to new institutions with limited data represent an important direction for practical deployment.

Third, interpretability and explainability requirements for clinical tools would necessitate methods for understanding which features drive specific predictions. The current black-box neural network provides limited insight into decision-making processes, which could hinder clinical adoption and trust.

Fourth, regulatory and validation requirements for clinical tools would necessitate substantially larger multi-center studies with prospectively defined endpoints and rigorous external validation. The present study represents an early-stage technical development rather than a deployment-ready system.

### 4.7 Future work

Building on these encouraging results, several clear opportunities exist to strengthen and extend this work. Validation across multiple independent COPD cohorts to assess generalizability, as well as broader benchmarking against alternative ordinal formulations. Incorporating richer clinical data such as imaging, laboratory values, symptom scores, and longitudinal follow-up would enable more comprehensive modeling of disease severity and progression. Further development of interpretability methods will be essential for clinical transparency and trust. Ultimately, prospective evaluation within real clinical workflows will be necessary to assess practical utility. Extending beyond GOLD stages toward refined phenotyping and COPD subtypes also represents a promising avenue to better capture disease heterogeneity and enhance clinical relevance.

## 5 Conclusion

We developed and evaluated an ordinal neural network with shared-private encoder architecture for COPD severity prediction across heterogeneous clinical datasets. The model achieved strong ordinal agreement (QWK = 0.894) and predominantly adjacent-stage errors on a validation set, outperforming standard multiclass classification and simpler baselines. Ablation studies demonstrated that both shared and private encoder components contribute meaningfully to performance.

The findings suggest that ordinal classification frameworks and shared-private architectures represent promising approaches for clinical severity assessment with heterogeneous data, but substantial additional validation is required to establish clinical utility. Future work should prioritize multi-center validation, systematic baseline comparisons, integration of richer data sources, and prospective evaluation in real clinical workflows. The present study provides initial technical proof-of-concept while clearly acknowledging the gap between experimental results and clinical deployment.

## Data Availability

The original contributions presented in the study are included in the article/supplementary material, further inquiries can be directed to the corresponding author.
